# Decision-making about palliative sedation for patients with cancer: a qualitative study in five European countries linked to the Palliative sedation project

**DOI:** 10.1186/s12904-024-01612-2

**Published:** 2024-12-21

**Authors:** Michael Van der Elst, Sheila Payne, Maria Arantzamendi, Nancy N. Preston, Ian Koper, Alazne Belar, Holger Brunsch, Séverine M. Surges, Claudio Adile, Yasmine Grassi, Zoe Cockshott, Jeroen Hasselaar, Johan Menten, Palliative Sedation Consortium, Michael Van der Elst, Sheila Payne, Maria Arantzamendi, Nancy N. Preston, Ian Koper, Alazne Belar, Holger Brunsch, Séverine M. Surges, Claudio Adile, Yasmine Grassi, Zoe Cockshott, Jeroen Hasselaar, Johan Menten

**Affiliations:** 1 Department of Oncology, Laboratory of Experimental Radiotherapy, Katholieke Universiteit Leuven, Herestraat 49, 3000 Louvain, Belgium; 2 International Observatory On End of Life Care, Faculty of Health and Medicine, Lancaster University, Lancaster, LA1 4AT UK; 3 Institute for Culture and Society-ATLANTES, Universidad de Navarra, Calle Universidad, 6, 31009 Pamplona, Navarra Spain; 4 IdISNA- Instituto de Investigación Sanitaria de Navarra. Palliative Medicine, Pamplona, Spain; 5 Department of Anesthesiology, Pain and Palliative Medicine, Radboud University Medical Centre, Geert Grooteplein 10, 6500 HB Nijmegen, Netherlands; 6 Department of Palliative Medicine, University Hospital Bonn, Venusberg Campus 1, 53127 Bonn, Germany; 7 La Maddalena Cancer Center, Via San Lorenzo 312, 90146 Palermo, Italy; 8 Department of Primary and Community Care, Radboud University Medical Centre, Geert Grooteplein 10, 6500 HB Nijmegen, Netherlands

**Keywords:** Palliative Care, Palliative Sedation, Decision-making, Qualitative research, Europe, Cancer, Health personnel

## Abstract

**Background:**

Palliative sedation refers to the proportional use of titrated medication which reduces consciousness with the aim of relieving refractory suffering related to physical and psychological symptoms and/or existential distress near the end of life. Palliative sedation is intended to be an end of life option that enables healthcare professionals to provide good patient care but there remains controversy on how it is used. Little is known about decision-making processes regarding this procedure. The aim of this study was to explore decision-making processes in palliative sedation based on the experiences and perceptions of relatives and healthcare professionals.

**Methods:**

We conducted a qualitative interview study with dyads (a bereaved relative and a healthcare professional) linked to 33 deceased patient with cancer who had palliative sedation, in seven in-patient palliative care settings in five countries (Belgium, Germany, Italy, the Netherlands, and Spain). A framework analysis approach was used to analyse the data.

**Results:**

Two main themes are defined: 1) Decision-making about palliative sedation is a complex iterative process, 2) Decision-making is a shared process between the patient, healthcare professionals, and relatives. Decision-making about palliative sedation appears to follow an iterative process of shared information, deliberation, and decision-making. The patient and healthcare professionals are the main stakeholders, but relatives are involved and may advocate for, or delay, the decision-making process. Starting palliative sedation is reported to be an emotionally difficult decision for all parties.

**Conclusions:**

As decision-making about palliative sedation is a complex and iterative process, patients, relatives and healthcare professionals need time for regular discussions. This requires a high level of engagement by healthcare professionals, that takes into account patients’ wishes and needs, and helps to facilitate decision-making.

**Supplementary Information:**

The online version contains supplementary material available at 10.1186/s12904-024-01612-2.

## Background

Clinical decision-making regarding the management of refractory suffering near the end-of-life crucially relies on goals of care, patients’ prognoses and, where available, advance care plans. Palliative sedation refers to the proportional use of titrated medication which reduces consciousness with the aim of relieving refractory suffering related to physical and psychological symptoms and/or existential distress near the end of life [1]. The delivery of sedation may be intermittent to provide respite for episodes of suffering, or may be continuous unto death. The effects of sedation may be light or deep. The practice of palliative sedation raises important clinical, ethical, social and moral concerns, and has been the focus of considerable controversy and debate [2, 3].

Medical decision-making refers to treatment related choices. Styles of decision-making may vary across cultures and countries due to many factors including the balance in preferences for individual versus relational autonomy. Research has identified three basic dimensions in end-of-life treatment that vary culturally: communication of diagnosis and prognosis; locus of decision-making; and attitudes toward advance directives and end-of-life care [4]*.* Typically, physicians have made medical decisions that are communicated to patients and/or families. Latterly, shared decision-making, where physicians present information to patients and families and discuss options, has been advocated [5–9]. One way to do this involves advance care planning which seeks to elicit patients’ preferences and wishes about end of life care [10]. This may be useful if the patient subsequently loses the capacity to express themselves and cognitive awareness.

To ensure best clinical practices regarding palliative sedation, several national and international guidelines have been developed in Europe [1, 11–13]. For instance, all guidelines refer to informed consent as a requirement for palliative sedation, a short life expectancy in case of deep and continuous palliative sedation, and the criteria of refractoriness of symptoms [1]. Guidelines address the decision-making process for palliative sedation and suggest that where possible the patient should be involved in the decision-making process and give informed consent [1]. How relatives should be involved in the decision-making process is less clear. Some guidelines suggest eliciting informed consent from relatives when the patient lacks mental capacity, while others indicate their active involvement during the decision-making process [1, 11–13]. When patients lack mental capacity, several guidelines suggest seeking consent of the relative or legal representative, while other guidelines indicate that it is the attending physicians’ decision [1, 11–13]. Little research has investigated how the decision-making process occurs in clinical practice, and relatives are rarely involved in these studies [14–18].

## Methods

### Aim, research question and design

We aimed to explore accounts of decision-making processes about palliative sedation, to determine how relatives and healthcare professionals perceived their involvement. We persued the resesearch question: How do bereaved relatives and healthcare professionals describe the process of decision-making related to starting palliative sedation?

We employed a qualitative approach using a multiple-case study design. We selected case study methodology because of its appropriateness for real-world situations, with in-depth investigation of specific contexts, where researchers cannot control confounding or other variables [19]. A case consisted of one bereaved relative and one healthcare professional closely involved in the use of palliative sedation for that deceased patient.

### Setting of the study

This study is part of the ‘Palliative sedation’ project which aims to explore the use of palliative sedation across five European countries (Belgium, Germany, Italy, the Netherlands, and Spain) for patients with cancer requiring sedation in specialist palliative care in-patient settings. Patients, relatives and healthcare professionals involved in the use of sedation were recruited to a study evaluating its effectiveness [20]. A qualitative interview study explored the experiences of bereaved relatives and healthcare professionals linked to deceased patients, involved in the decision-making process [21].

### Population and sampling

The sample was identified from ten patients in each country who were treated with palliative sedation before death in a concurrent observational cohort study [20]. The type of sedation could be light or deep, intermittent or continuous depending upon the needs of the patient and the clinical practices in each setting. Eligible participants had to be ≥ 18 years and speak the local language. Only bereaved relatives of patients participating in the observational study were eligible [20]. A physician or nurse involved in the care of that deceased patient who received palliative sedation was eligible. Interviews were held 2–3 months after the sedated patient died. Recruitment took place from July 2021 to June 2023.

### Data collection for bereaved relatives and healthcare professionals

**Fig. 1:**
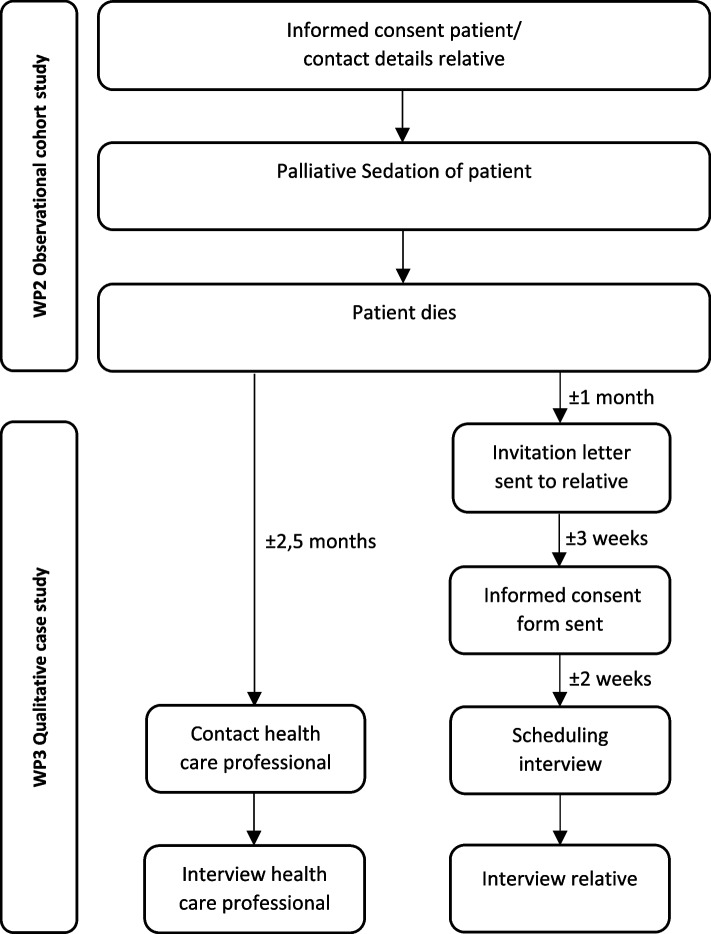
Flow chart of recruitment of participants

One month after the patient’s death, a closely involved relative was invited to participate in the study. A healthcare professional closely involved in the care of the same sedated patient was contacted. Those expressing interest were offered the opportunity to discuss the study and arrange an interview. Details of recruitment procedures are documented in the study protocol [21] and shown in Fig. 1.

Semi-structured interviews were selected as they offer participants the possibility to discuss new topics and/or the interviewer to probe deeper into a specific topic; while the predetermined topic guide ensures a certain content homogeneity across interviews [22, 23]. An interview guide was developed based on the original European Association for Palliative Care (EAPC) framework for palliative sedation [11]. The interview guide included the following topics: 1) initiation and information on palliative sedation, 2) deliberation and decision-making. Healthcare professionals were also asked about refractory symptoms (Supplementary file 1: Interview guide). The interview guide was pilot-tested with two nurses.

Training was given to all researchers (MVDE, IK, AB, MA, HB, SS, YG) conducting interviews prior to starting data collection. Two of the interviewers have a medical background (eg. Nurse, physician) the others had no medical background (eg., social scientist). The interview took place at the interviewee’s preferred place: at home, clinical centre, or online, in their local language. Interviews were digitally recorded and participants were informed that the recording could be stopped upon request. The informed consent form was signed before the start of the interview (see Fig. 1). The healthcare professional could use the patient’s medical records, if necessary, to aid recall.

The audio-recorded data were transcribed verbatim into local languages, pseudo-anonymized, inputted into NVivo (version 12), and then audio-recordings were destroyed. Participants and settings were allocated a numerical code to ensure anonymity. Researchers wrote field-notes about each case to provide contextual information.

### Data analysis

**Fig. 2:**
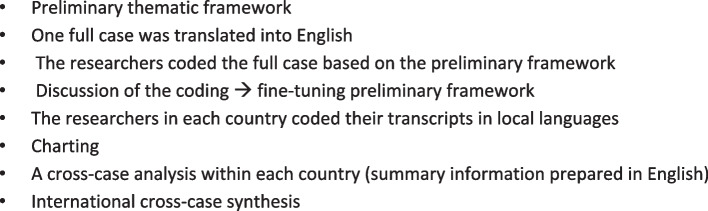
Flow chart of analysis

The analysis was conducted in two phases: 1) initial analysis of data in local languages, and 2) an international analysis using summary information (field notes) and selected quotes translated into English. See Fig. 2 for details.

A framework analysis approach was used [22, 23]. A preliminary thematic framework was developed based upon previous research and the interview guides. The first full case was charted by all researchers in the five countries according to the framework. Initial meetings discussed the application of the framework and made adjustments. Then all researchers undertook the analysis of their local transcripts (in their original language) based on the adapted framework (Supplementary file 2). This combined an analysis of summary information to answer our research aim and research question across multiple cases, settings and countries. Direct quotations have been selected to highlight typical responses, and are indicative of the diversity reported. To establish rigor, we adhered to Consolidated Criteria for Reporting Qualitative Research (COREQ) guidelines in reporting [24].

## Results

### Description of the sample

We start by describing the seven in-patient settings, located in five countries (see Table 1), and the individual participants linked to deceased patients in the 33 cases (see Table 2), before presenting the findings. All sites provided specialist palliative care delivered by multidisciplinary teams. We interviewed *n* = 18 spouses, *n* = 13 adult children, and *n* = 2 siblings. The majority were female *n* = 21, male *n* = 12. We interviewed *n* = 17 physicians, *n* = 15 nurses and *n* = 1 nursing assistant. Most had between 5- < 15 years of experience in palliative care and most were female (*n* = 29). The average duration of an interview with a relative was 45 min and with a healthcare professional 30 min. The average age of the patients was 68 years, the relatives had an average of 57 years, and the healthcare professionals were on average 48 years old.

**Table 1:** Characteristics of the seven settings

	Setting 1	Setting 2	Setting 3	Setting 4	Setting 5	Setting 6	Setting 7
Country (region)	Belgium (Flanders)	Germany (North Rhine-Westphalia)		Italy (Sicily)	Netherlands (Gelderland)		Spain (Navarra)
Setting(s)	Palliative care unit (PCU) in the university hospital	Palliative care unit in the university hospital	Palliative care unit in the hospital	Hospice in the hospital	Hospice in the middle of a city (not in a hospital)	Hospice in a rural area (not in a hospital)	Palliative Care team is part of the university Hospital
	12 rooms/12 beds	6 rooms/8 beds	6 rooms/8 beds	10 rooms/10 beds	10 rooms/10 beds	8 rooms/8 beds	No fixed number of beds
	No artificial food or hydration	Artificial food and hydration when indicated	Artificial food and hydration when indicated	Artificial food and hydration on request of the family	No artificial food or hydration	No artificial food or hydration	Artificial food and hydration when indicated
Patient referral criteria	Life expectancy < 3 months	There is no admission restriction based on life expectancy	There is no admission restriction based on life expectancy	Life expectancy < 3 months	Life expectancy < 3 months	Life expectancy < 3 months	There is no admission restriction based on life expectancy
	Most patients came from the hospital to the PCU, but some patients came directly from home care	Most patients came from the hospital to the PCU, but patients also came directly from home care	Most patients came from the hospital to the PCU, but patients also came directly from home care	Most patients came from the hospital to the PCU or hospice, but patients also came directly from home care	Most patients came from the hospital to the hospice, but patients also came directly from home care	Most patients came from the hospital to the hospice, but patients also came directly from home care	Most patients are referred to the palliative care support team by the hospital oncologist. Some patients may come from home

Key: *PCU* Palliative care unit

**Table 2:** Characteristics of deceased patients, bereaved relatives and healthcare professionals

		Deceased Patient		Bereaved Relative		Healthcare professional			
Case	Country/ (Setting)	Age range	Gender	Affiliation patient	Age Range	Occupation	Age range	Gender	Professional experience (years)
1	Belgium (1)	65–70	Male	Daughter	30–35	Nurse	45–50	Female	> 15
2	Belgium (1)	65–70	Female	Daughter	30–35	Nurse	50–55	Female	> 15
3	Belgium (1)	85–90	Female	Daughter	50–55	Nurse	55–60	Female	5–15
4	Belgium (1)	65–70	Male	Sister	60–65	Nurse	50–55	Female	5–15
5	Belgium (1)	75–80	Female	Husband	75–80	Physician	25–30	Female	< 5
6	Belgium (1)	75–80	Female	Son	55–60	Nurse	50–55	Female	> 15
7	Belgium (1)	65–70	Female	Husband	70–75	Nurse	60–65	Female	5–15
8	Belgium (1)	85–90	Male	Daughter	65–70	Nurse	30–35	Female	5–15
9	Germany (2)	65–70	Female	Husband	60–65	Nurse	60–65	Female	> 15
10	Germany (2)	55–60	Male	Wife	60–65	Physician	40–45	Female	> 15
11	Germany (2)	80–85	Female	Daughter	55–60	Nurse	55–60	Female	> 15
12	Germany (3)	80–85	Male	Wife	80–85	Physician	60–65	Female	> 15
13	Italy (4)	75–80	Female	Husband	70–75	Physician	40–45	male	5–15
14	Italy (4)	45–50	Female	Husband	50–55	Physician	30–35	Female	< 5
15	Italy (4)	45–50	Female	Husband	50–55	Physician	40–45	male	5–15
16	Netherlands (5)	45–50	Female	Husband	50–55	Nurse	35–40	Female	5–15
17	Netherlands (5)	75–80	Male	Wife	70–75	Physician	50–55	Female	> 15
18	Netherlands (5)	70–75	Female	son	40–45	Physician	50–55	Female	> 15
19	Netherlands (5)	60–65	Male	Wife	55–60	Nurse	35–40	Female	5–15
20	Netherlands (5)	85–90	Male	Daughter	30–35	Physician	50–55	Female	> 15
21	Netherlands (6)	85–90	Female	Son	60–65	Physician	30–35	Female	< 5
22	Netherlands (6)	75–80	Female	Daughter	50–55	Nursing-assistant	45–50	Female	> 15
23	Netherlands (6)	65–70	Female	Daughter	40–45	Physician	40–45	Female	> 15
24	Spain (7)	35–40	Male	Wife	40–45	Physician	40–45	Female	5–15
25	Spain (7)	65–70	Male	Wife	65–70	Nurse	40–45	Female	> 15
26	Spain (7)	55–60	Male	Wife	55–60	Physician	25–30	Female	< 5
27	Spain (7)	55–60	Female	Husband	60–65	Physician	35–40	Female	5–15
28	Spain (7)	55–60	Male	Wife	X	Physician	35–40	Female	< 5
29	Spain (7)	60–65	Female	Daughter	35–40	Nurse	55–60	Female	5–15
30	Spain (7)	65–70	Male	Wife	X	Nurse	55–60	Female	> 15
31	Spain (7)	75–80	Female	Brother	X	Nurse	55–60	Female	5–15
32	Spain (7)	75–80	Male	Wife	70–75	Physician	40–45	Male	5–15
33	Spain (7)	75–80	Female	Daughter	45–50	Physician	60–65	Male	> 15

KEY: X indicates missing data, data about age range must be interpreted as [x, y]

### Analytic themes

Our findings are structured in two main themes: 1) decision-making as an iterative process, and 2) a process of shared decision-making.

## Decision-making as an iterative process

**Fig. 3:**
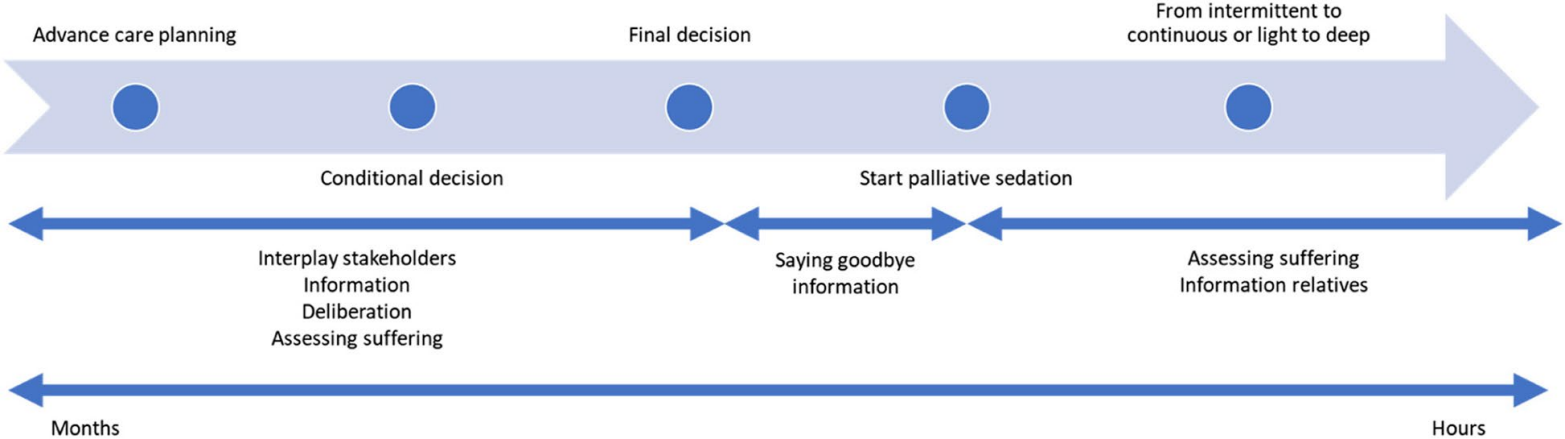
Iterative process of decision-making in palliative sedation

Participants in most cases often emphasised that decision-making regarding palliative sedation did not occur as a single event, but was a dynamic and iterative process occurring over time. In most cases, once the idea of palliative sedation had been introduced, there was an ongoing process of information-sharing, observation, and deliberation between patients, relatives, and healthcare providers until a decision was made. This process generally followed a sequence of starting the conversation, a conditional decision (where prior decisions were made to be enacted in specific circumstances), followed by a final decision to proceed with palliative sedation (see Fig. 3).

### How the conversation started

Several bereaved relatives reported that the idea of palliative sedation was initiated a while before the phase of dying (in one case a year before death). The concept of sedation was often introduced in a broader conversation about end-of-life care triggered by previous experiences or during a hospital admission during the illness trajectory. In these cases, patients and families had detailed knowledge of their condition and prognosis and a clear view of how they would like to proceed including prior consideration of palliative sedation.

*“I think two years ago, mum and dad went together to the palliative physician. Mum had*

*everything written on paper, her Do-Not-Resuscitate -code ... she had everything on paper and afterwards she also let us know …”(Belgium, case 2, relative)*

According to the bereaved relatives, while some patients and families had an understanding of their health condition and prognosis, they were unaware of what could be offered as palliative care options. In a few cases, it appeared that patients and families came to palliative care services without adequate awareness of their prognosis, with some even hoping for further cancer treatment and cure.

*“There are people who say: "well, this is it, this is the end, this is the end here and now", but he was always thinking that he was going to get better, and it was Easter, and he was Catholic, and then he would look at me and say: "I'm waiting for my miracle", and the next day he would say to me: "I'm still waiting for my miracle": "I'm still waiting for my miracle". (Spain, case 30, relative)*

Between the settings, there were different approaches to providing information to the patient. So, while in the Netherlands settings, palliative sedation was addressed proactively, in other settings, palliative sedation was mostly discussed reactively after it was initiated by the patient (eg. Site 1). Some healthcare professionals explicitly avoided reference to “palliative sedation”, but used terms such as ‘comfort’ or ‘relief’ (eg. Site 7), while in the Netherlands, Belgium and Germany palliative sedation is explicitly named in conversations with patients and families.

*“There were conversations about there being no more treatments, and indeed, we talked to him about there being nothing more to do, that we needed to focus on comfort” (Spain, case 31, health care professional)*

Both bereaved relatives and healthcare professionals reported that in end-of-life conversations, pros and cons of palliative sedation, and the concerns of patient and relatives were discussed. Several healthcare professionals suggested that information frequently needed to be repeated in subsequent meetings since not all information was understood initially, because of the emotional distress of patients and families.

*"The options have been repeatedly explained, not once but a whole number of times. I thought at least twice by myself and questioned again to the patient. It has been proven that in emotional conversations, we only remember a small percentage of the information. So, the repetitiveness is very important for clarity." (Belgium, case 1, healthcare professional)*

### Conditional decision

According to bereaved relatives, a few patients were initially reluctant to discuss end-of-life care, although most patients and families were open to address this topic. Bereaved relatives and healthcare professionals reported that the expected worsening of the patient’s medical condition with more suffering was often a concern. Anticipating this, many patients formulated a conditional decision, that if the situation worsened, then they wanted to be sedated. This ‘conditional decision’ was in many cases a strong directive on how to proceed. In most of these cases, relatives supported patients’ decisions about palliative sedation.

*“It was discussed in advance that she would like this [palliative sedation] in case her suffering would become intolerable, and she was probably very, very restless throughout the weekend, always trying to get up and then it was discussed with her that palliative sedation could be started now and she agreed to this.” (Germany, case 11, healthcare professional)*

### Decision to start sedation

Data suggests that following initial conversations and conditional decision-making, the final decision to proceed with palliative sedation can be complex, and emotionally difficult for patients and families. This final decision was often precipitated by a deterioration in the patient’s condition, and the presence of refractory suffering, prompting further discussions about commencing palliative sedation. In some cases, this was initiated by the healthcare team, after a period of observing the patient’s symptoms and team discussions. In other cases, it was initiated by a verbal request or non-verbal communication from the patient. Our findings suggest that whilst families tended to be influenced mainly by observing patients’ physical symptoms, healthcare professionals were more likely to also consider psychological and existential suffering as indications for palliative sedation.

*“When she started to have these fears for her end of life, we started talking about sedation, how to modulate sedation, so we shared the process with her. At a certain point, when she couldn't take it anymore, we started discussing it during the visit and said to each other: let's start the sedation because perhaps the time has come to control things better.” (Italy, case 13, healthcare professional)*

A final decision about palliative sedation usually involved further discussions between all parties, often referring to previous conversations. When the patient was no longer able to communicate, any earlier conditional decision was a valuable directive, along with consultation with the relatives. However, making the final decision to start palliative sedation was often very difficult and emotionally distressing for patients and families, even when patients were initially committed to it. In a few cases, ambivalence was reported when patients and families struggled between reducing suffering and saying their final goodbyes. When tensions increased during this period, the healthcare professionals reported that they mediated to reach a consensus.

*“He was suffering from these five things, and he indicated that it was enough, and he pointed to his mouth again, meaning I want a sip, I’m thirsty. At that time, I started the conversation with his daughters like, I think we need to start palliative sedation now because this is unbearable for him. And his daughters really struggled with that. We had this conversation on Monday, and they had to sleep on it for a night, so Tuesday we had the conversation again, now with all daughters present. A long conversation, even though you would think, this is clear as day, that man is suffering terribly, and he doesn’t have much longer to live. Then we talked for about an hour and at some point one of the daughters said: maybe giving him palliative sedation is the merciful thing to do. Yes, I confirmed that obviously, and then they were all aboard like, okay this is our decision. That evening, half past seven we started sedating.” (Netherlands, case 20, healthcare professional)*

When light or intermittent sedation was insufficient, additional decision-making was precipitated. Patients who were still conscious were involved, otherwise, physicians took the lead after informing or consulting with relatives.

## Process of shared decision-making

**Fig. 4:**
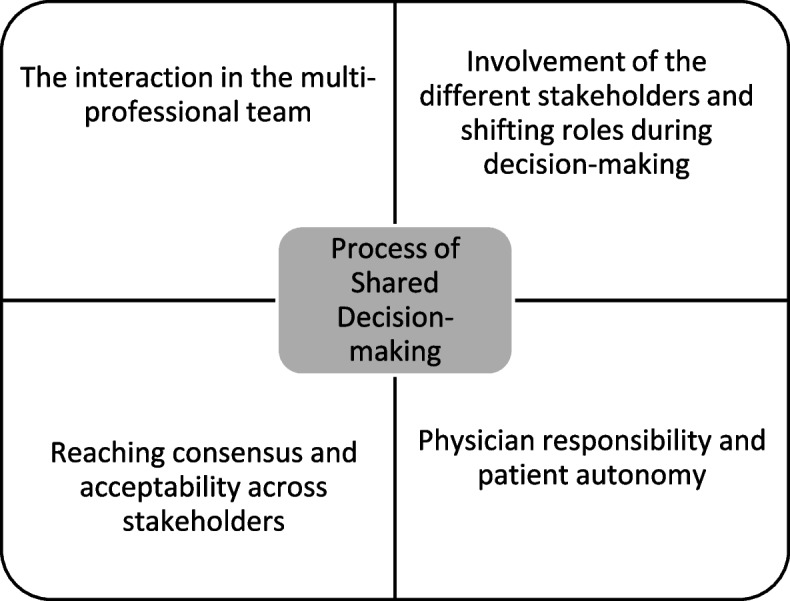
Process of shared decision-making

The decision-making process involved the patient, relatives, and healthcare professionals in all cases and in all settings, and appeared to be dynamic and interactive (Fig. 4). There were many conversations between the different stakeholders.

*“... then practically, together with her husband, she demanded it [palliative sedation] herself, or we suggested it and they then demanded it. But they were already fully aware of what this palliative sedation meant. So, in this case it was a kind of intertwined deliberation.” (Germany, case 9, healthcare professional)*

### The interaction in the multi-professional team

Generally, physicians led decision-making about palliative sedation. However, they emphasised that their decisions reflected the views of the patient, relatives, and caring team. The nurses’ input was particularly emphasised as they were closely involved in hands-on caring. During multidisciplinary team meetings and informal contacts with colleagues and relatives, the status of the patient was repeatedly discussed. When their condition deteriorated or they requested palliative sedation, it was most often nurses who informed the physician.

*“The doctor has the final responsibility. But the decision to start sedation is made together, also with the patient. And we, the healthcare team, we see signs, we see how someone is feeling and we can discuss this with them, amongst ourselves and with the doctor. This cooperation is a beautiful thing. […] And family is also very important, they can also indicate they see changes with the patient. Maybe even things we don’t see.” (Netherlands, case 16, healthcare professional)*

### Involvement of the different stakeholders and shifting roles during decision-making

Participants indicated that different stakeholders were constantly exchanging information and deliberating about palliative sedation. Both the patient and healthcare professionals were always involved in the decision to start palliative sedation. Relatives appeared, in general, to be less proactive during decision-making, mainly supporting others decisions. However, roles could vary over time. For instance, if the patient became unable to communicate, the healthcare professional took the lead and relatives were more actively involved in the decision. In some cases, the relative took a more active stance by advocating for the patient.

*“When we proposed sedation more than once during the day...the patient said no. The daughter said “whatever my mother says”. But when the daughter was seeing the situation and getting very stressed, she would start, "Can't you do anything? I just don't know what...". We would explain again that medically we could sedate her (patient), but the patient didn't want it... and the daughter would respond, "Oh, right." The daughter had a hard time handling that situation”. (Spain, case 29, healthcare professional)*

While in other cases, it was reported that the relatives delayed the start of sedation because they were struggling with saying goodbye.

*“The decision was only about the moment when the palliative sedation would be started. I*

*was the one who retarded the longest, how should I say, who delayed everything. I didn't want*

*to lose her.” (Belgium, case 5, relative)*

### Reaching consensus and acceptability across stakeholders

Several healthcare professionals indicated the importance of consensus between everyone regarding sedation. Sometimes delays were linked to patients’ unreadiness to die or relative’s reluctance to proceed; occasionally unexpectedly after agreement with the conditional decision. Healthcare professionals reported that these situations could cause tensions. In most cases, a multidisciplinary team meeting or a moral case deliberation was organized to address concerns. Healthcare professionals perceived that good care was provided when patient’s wishes were respected.

*“With him [patient] too, it was very easy because after having talked everything over, he saw the moment very clearly. We even offered him if he wanted his mother to come to say goodbye, who was an hour away; he said no, that they had already talked everything over and that he clearly saw that it was the time. His wife is a very strong woman who supported him in the decision at all times and supported him, and who really appreciated the rest we gave him.”(Spain, case 24, healthcare professional)*

### Physician responsibility and patient autonomy

Usually the physician took responsibility for the final decision about sedation, drawing upon the input of the healthcare team and patient preferences. It appears that most bereaved relatives supported the position taken by the patient, as in this example.

*“Yes of course, she always decided for herself, I gave my opinion but she (patient) decided everything. In these matters, one cannot make decisions for others.” (Italy, case 13, relative)*

The data suggest that relatives wanted to be in a supportive role and not in a decision-making role.

## Discussion

In this qualitative study, we explored decision-making processes in palliative sedation based on accounts from bereaved relatives and healthcare professionals elicited in 33 cases, in five European countries. The results show that decision-making was an iterative and dynamic process of information giving, deliberation, with conditional decisions being made by some patients, and gradually evolving agreement on a final decision. The results also suggest that palliative sedation is a process of shared decision-making involving the patient, healthcare professionals, and relatives. During the process, interaction between stakeholders shifted with healthcare professionals seeking a consensus.

Shared decision-making is advocated in professional–patient interaction [6]. However, the concept of shared decision-making is rather vague, with some integrative models proposed [7–9]. Shared decision-making may include common elements such as patient values and preferences, presenting several options, mutual agreement, and information exchange [7]. The UNBIASED study reported that there was considerable variation in the role played by the patient in the decision-making. At one end of the spectrum, decision-making was primarily physician-driven. At the other end of the spectrum, the patient was the decision-maker while the physician’s role was informing the patient, evaluating whether, and when, the patient’s condition fulfilled the medical criteria [15]. We found that relatives across all settings valued the opportunity to be involved in end-of-life decision-making and taken into account the patient preferences. Based on our data, many of these elements were reported, for instance, the importance of the patient's preferences. Healthcare professionals highlighted the benefits of joint discussions with patients, and the importance of multidisciplinary team meetings in facilitating agreements on palliative sedation. It seems that shared decision-making is a feasible approach to decision-making about palliative sedation [6]. However, the specific end-of-life context of palliative sedation requires an open awareness of dying which may be problematic in some cultures [25, 26]*.*

Decision-making in palliative sedation was often described as a process with different moments of initiation, information, deliberation, criss-crossing each other. This complex iterative process with regular conversations was noted in previous research [15]. We found that some patients had end-of-life conversations in advance, before entering the palliative care setting [10], thereby increasing their awareness of end-of-life options. An international clinical trial of advance care planning for patients with advanced cancer in hospital indicated that its use was associated with palliative care input [27]. Our findings contrast with evidence from a systematic review that indicated decision-making seemed to be performed late in the disease trajectory [18]. This might be explained by studies focusing on decision-making only at the start of sedation, rather than over time. For example, there is a distinction between the decision of whether to use sedation and the decision about the timing of sedation [15]. Most patients in our study made conditional decisions in advance, which are important directives for the healthcare team and relatives. In some cases, this conditional decision was formulated a long time before the start of palliative sedation. Since undertaking our study, revised recommendations from the EAPC highlight the importance of advance care planning, better communication with relatives and support within the multidisciplinary team [1].

### Strength and limitations

Through detailed analysis of accounts of decision-making, it was shown how decision-making interactions were carried out between all participants. Our approach facilitated identification of patterns during the analysis, facilitated by numerous research team meetings to enhance accuracy in interpretation and validation of findings. The Covid-19 pandemic impacted recruitment to the cohort study [21], and meant some interviews were conducted online, where necessary. Translation of interview excerpts rather than full transcripts may have reduced nuanced understandings of specific contexts but frequent research meetings throughout the project may have mitigated this. Our study focuses only on cancer patients in specialised palliative care settings and may lack transferability to other settings. Some healthcare professionals had difficulty recalling the specific details of the deceased patient after three months.

#### Implications and recommendations

Our study shows that the decision for palliative sedation is not a single-moment event but a process, in which information is given repeatedly and with constant interaction between the stakeholders. It’s because of this iterative process that all stakeholders can gradually reach a consensus. This iterative process facilitates shared decision-making, which we recommend. We recommend advance care planning conversations, so patients and relatives are well informed and conditional decisions can be made and used as a directive when a sudden intervention is needed. We also recommend the use of multidisciplinary team meetings or moral case deliberations to address difficult ethical dilemmas. These recommendations are based on data across different countries and settings. However, our data suggest also differences in approach to initiate a sensitive topic such as palliative sedation. Consequently, guidelines should take cultural differences into account.

## Conclusions

Our research has illustrated the complexity of decision-making associated with initiating palliative sedation for patients with advanced cancer in-patient care in five European countries. Decision-making about palliative sedation involves an iterative dynamic process predominantly between patients and healthcare professionals. The accounts of relatives and healthcare professionals largely indicated that the decision is a shared process. Our findings indicate that healthcare professionals need to address palliative sedation as a potential end-of-life care option to deal with refractory symptoms, earlier rather than later in the disease trajectory. Advance discussion of end-of-life options increases awareness of patient preferences often resulting in directives (based on conditional decisions). Future research should focus on when, and how, healthcare professionals should discuss palliative sedation and other end-of-life care options with patients (and relatives). Future research should also explore decision-making within the multidisciplinary team and use of sedation in other care settings.

## Supplementary Information

Supplementary Material 1. Interview guide.

Supplementary Material 2. Code book.

## Data Availability

The datasets generated and/or analysed during the current study are not publicly available due the protection of the traceability. A synthesis of each transcript will be made accessible for third party re-use on DANS-EASY after the project is finished. Data are however available from the corresponding authors upon reasonable request.

## Abbreviations

COREQ: Consolidated Criteria for Reporting Qualitative Research
EAPC: European Association for Palliative Care
