# Supplementary material for: Decision-making about palliative sedation for patients with cancer: a qualitative study in five European countries linked to the Palliative sedation project
Source: BMC Palliat Care. 2024 Dec 21;23:295. doi: 10.1186/s12904-024-01612-2 (PMC11662527; doi:10.1186/s12904-024-01612-2)
Supplement: Supplementary file 1 — Supplementary Material 1. Interview guide. [file 12904_2024_1612_MOESM1_ESM.docx]

**Supplementary file 1: Interview guide**

| Table S1: Topic guide and interview guide: relatives of the patient | |
| --- | --- |
| *Topics* | Sample questions: probes and prompts |
| *1. Initiation and information on Palliative Sedation* | -How did the idea of sedation arise?  -What were your initial thoughts about palliative sedation?  -How did you feel when the application of sedative medication was discussed for the first time?  -How did your relative react to this discussion? Was this a relief, made this him/her more anxious?  -Can you tell me more about the things that were discussed during this conversation(s)?  -What information about palliative sedation/end-of-life did you receive?  -What information about palliative sedation/end-of-life did you want/needed? |
| *2. Deliberation and Decision-making process of palliative sedation* | -Can you tell me the reasoning why palliative sedation was chosen?  -Which alternative therapies were discussed (to treat the symptoms?)  -Can you describe how the decision about palliative sedation was made?  -Which persons were involved in the decision-making process and why? |
|  | -Would it be ok if I ask you to tell me how you experienced this period?  -Can you tell me more about the interaction between the family members/relatives of a patient and the health care professionals at this moment? |

| Table S2: Topic guide and Interview guide for Health care professionals | |
| --- | --- |
| *Topic list* | Sample questions: probes and prompts |
| *1. Initiation and information on Palliative Sedation* | -Thinking about patient X, how did the idea of sedation arise?  -How would you describe palliative sedation?  -What were your initial thoughts about palliative sedation in the case of patient X?  -When palliative sedation was mentioned the first time with patient X, what was discussed with the patient (relatives of the patient)?  -How did patient X react/respond to the idea of palliative sedation?  -How did the relatives of the patient react/respond to the idea of palliative sedation  -Can you tell me more about the things that were discussed with the family during the initiation of PS  -Which information was requested by the patient/relatives of the patient?  -How was the interaction with the other health care professionals at this stage of the PS |
| *2. Refractory symptoms* | -Thinking about patient X, which refractory symptoms were present?  -Thinking about patient X, how was the assessment of the refractory symptoms done?  -Did the patient suffer existentially? How was this assessed? How did you notice this existential suffering? |
| *3. Deliberation and decision-making of palliative sedation* | -Can you tell me why, in the case of patient X, palliative sedation was chosen?  -Who was involved in the decision-making process?  -How was, in the case of patient X, the decision made?  -What was your role in the decision-making process?  -Were alternative therapies (to treat the symptoms), which? and why was in the end not for this therapy chosen?  -How was the interaction with the family members at this stage of the procedure?  -How was the interaction with the other health care professionals at this stage of the PS?  -How did you experience this decision-making process? |
