# Supplementary material for: Decision-making about palliative sedation for patients with cancer: a qualitative study in five European countries linked to the Palliative sedation project
Source: BMC Palliat Care. 2024 Dec 21;23:295. doi: 10.1186/s12904-024-01612-2 (PMC11662527; doi:10.1186/s12904-024-01612-2)
Supplement: Supplementary file 2 — Supplementary Material 2. Code book. [file 12904_2024_1612_MOESM2_ESM.docx]

**Supplementary file 2: Code book**

| **Categories** | **Framework/Codes** | **Clarification** | |
| --- | --- | --- | --- |
| Demographics | Clinical site |  | |
|  | Country |  | |
|  | Gender |  | |
|  | Relatedness |  | |
| Choice PS | Intermittent versus continuous |  | |
|  | Light versus deep |  | |
| Initiation PS | Initiation PS | Who initiated the idea of PS: HCP, patient, relative? What triggered it to discuss PS. Was PS something unknow or did the patient and relatives had previous experiences with PS? Did the patient had an advance care plan before initiation of PS? How did the respondent and the relatives react on the idea of PS? | |
|  | o   Presence Advance care plan |  |  |
|  | o   Previous experiences |  |  |
| Eligibility PS | Symptoms | What were the criteria used to determine whether the patient was eligible for PS? Did the participant suggest physical symptoms like pain, delirium, vomiting, dyspnoea to apply PS. Does the participant suggest psychological, social, existential suffering like: loss of dignity, fear, panic, death anxiety, dependency/inability to take care of oneself, hopelessness, wish to control the time of death by oneself, feeling of worthlessness, feeling of meaninglessness, loneliness, lack of social support, isolation, others? Were these symptoms refractory? How was/were the refractory symptom(s) measured/assessed/evaluated? Did one use objective measures (e.g., RASS) or subjective measured (based on the patient opinion, relative’s opinion, professional opinion). In case of subjective measures, previous research has shown that relatives overestimate symptoms, while HCPs underestimate symptoms. When determining the eligibility for PS, was this done in a formal way (e.g., meeting) are rather in an informal (e.g., in the corridor/hall informal conversations, individual or in group)? Behind closed doors or openly? With or without the input of the patient?  The HCP experience uncertainty concerning person life expectancy.  All other treatments were tried or discussed and the conclusion was that sedation was the last resort option. In this sub code chunks of text with regard to all the options they tried should be added. | |
|  | o   Refractory symptoms |  |  |
|  | - Physical |  |  |
|  | - Existential |  |  |
|  | o   Future suffering |  |  |
|  | o   Potential refractory symptoms |  |  |
|  | Assessment symptoms |  |  |
|  | Uncertainty: Life time |  |  |
|  | Alternative treatment | All other treatments were tried or discussed and the conclusion was that sedation was the last resort option. In this sub code chunks of text with regard to all the options they tried should be added. | |
| **Decision making** |  |  | |
|  | Role of patient in the decision-making process | Decision about patient not with patient | Looking for motivations why the role of the patient was not paramount (decision about them not with them), perhaps the patient was not in a state which made it possible to participate in the decision process (dementia, or others…). Lack of consensus, Lack of conversation, Lack of partnership, Lack of mutual agreement, Lack respect for autonomy |
|  |  | Shared decision making | Shared decision-making is not a decision made by the health care team nor a decision made by the patient. Shared decision-making is a mutual agreement by the patient AND the health care team (HCT). Both take responsibility for the decision. It is a partnership based on equality between both, the HCT can give their advice and make recommendations. Important terms are the presence of conversations (not debates or discussions), consensus, information etc. |
|  |  | Decision-maker |  |
|  | Role of family in the decision-making process | Facilitator for PS | Involvement of the relatives in the decision-making. The relatives can be a facilitator for PS, for instance can inform the patient about his options, open the conversation/communication with regard to PS or end of life, can be a liaison between patient and HCPs. The relative can also put pressure on the health care team and the patient to apply PS e.g., the burden and distress the relative is experiencing because of the suffering. The family members can be a barrier to apply PS, for instance religious beliefs, not ready to say goodbye, still in denial the patient will die soon. The relatives can be the decision-maker for instance in cases whereby the patient is unable to declare his wishes. The family members can also have no role in the decision for instance if the patient is able to declare his wishes or hospital policy to let the decision made by the patient or the HCPs. |
|  |  | Pressure to apply PS |  |
|  |  | o   burden/distress |  |
|  |  | Barrier to apply PS |  |
|  |  | o   religious beliefs |  |
|  |  | o   burden of the decision |  |
|  |  | Decision maker |  |
|  |  | No role |  |
|  | Role of the HCP in decision making process | Decision maker |  |
|  |  | No role in decision |  |
|  |  | The patient/family was (not) empowered to make a decision by HCP | Was the patient empowered to decide are not? Empowerment includes several elements: 1) Was the patient informed, was sufficient information given, had the patient sufficient access to information to decide. 2) Did the patient have the ability to make a choice, were several choices offered for instance the choice to intermittent or continuous sedation. 3) The patient was active involved in the decision the patient was enabled to give his honest opinion even if others disagreed, the patient could speak confidently with the health care team and the relatives, the patient could express strong feelings. 4) self-esteem: a person's overall sense of self-worth or personal value. In other words, how much do you appreciate and like yourself. It involves a variety of beliefs about yourself, such as the appraisal of your own appearance, beliefs, emotions, and behaviours. |
|  | Indecisiveness |  | |
|  | (Other) barriers decision making | Opinion health care prof  Legal considerations  Institutional policy  Moral conflicts  People who don’t understand the situation of the patient. Perhaps, the relative is still in denial and don’t acknowledge that the patient is dying. The relative could be confused because of the hectic of the situation. | |
|  | Pros and cons of PS are (not) fully shared with patient/family | Deliberations of the options are discussed with the patient (or the relatives), what are pros what are cons of certain choices for instance deep palliative sedation, a con is the patient will not be able to communicate anymore, pro the patient will have less pain/ will not be suffering anymore. -->discussing the pros and cons is often also given information (can be double coded) | |
| Control (or not) | Mastery/empowered/self-esteem/burden decision-making | Mastery refers to a person’s ability to control circumstances in his or her life (Pearlin & Schooler, 1978). The relative can experience the situation as not under control, not able the affect/change the situation. | |
| Concerns | Hasten death/double effect | The principle of double effect is a rule of conduct frequently used to determine when a person may lawfully and ethically perform an action from which two effects will follow, one bad, and the other good. Four Basic Components of the Principle of Double Effect | |
|  |  | a.       The nature of the act must be good or morally neutral and not in a category that is absolutely prohibited or intrinsically wrong. | |
|  |  | b.      The intent of the healthcare provider must be good, and while the good effect and not the bad effect must be intended, the bad effect can before seen, tolerated, and permitted. | |
|  |  | c.       A distinction between means and effects must be envisioned, in that death must not be the means to the good effect. In other words, the good effect must be produced directly by the action, not by the bad effect. Otherwise, the agent would be using a bad means to a good end, which is never allowed | |
|  |  | d.      A proportionality between the good and bad effects must be substantiated by reason, in that the good effect must exceed or balance the bad effect (Rousseau 2000, Quill 1997) i.e. the good effect must be sufficiently desirable to compensate for the allowing of the bad effect. (https://palliative.stanford.edu/palliative-sedation/ethical-framework-of-palliative-sedation-the-principle-of-double-effect/) | |
|  | Opportunity for wider family consultation | It happens that HCP only consult/inform a limited number of persons. Important, is there an explanation why? For instance, the urgency of the situation (patient is suffering a lot) which made it impossible to consult more family members; the standard practice of palliative sedation includes only consultation with the patient or the nuclear family. | |
|  | Families/patients feel alternatives (not) explored | Other medications were not discussed for instance fentanyl instead of morphine, new medication for instance anxiolytics. In case of existential suffering, consultation with chaplain/spiritual care, consultation with social worker, consultation with psychiatrist, consultation with psychologist, antidepressants. Different options were discussed/offered with regard to PS light versus deep, intermittent versus continuous. In Belgium and the Netherlands, was euthanasia discussed? | |
|  | Feelings the patient was forced into sleep |  | |
|  | Dying process un-naturally prolonged? |  | |
| the effect of the PS?  Suboptimal sedation | Effect of PS | What was the effect of the palliative sedation on the refractory symptoms of the patient. How is this assessed? Is the patient less consciousness or unconsciousness? In recent years, doubts have risen whether patients labelled ‘‘unconscious’’ really are completely insensate and unaware. Studies in different types of patients and settings that critically reviewed awareness have consistently reported that persons were, in contrast to what was assumed by the caregivers, not always (completely) unaware (deschepper et al.). Also, patients with locked-in syndrome may be mistakenly considered unconscious, as may some (rare) patients during general anaesthesia. Lock-in syndrome: (Acquired neuromuscular disorder characterized by complete paralysis of voluntary muscles and lower CRANIAL NERVES except for limited voluntary eye movements. It is due to various cerebrospinal disconnections at or near the PONS and the POSTERIOR CRANIAL FOSSA, typically secondary to pontine haemorrhage or infarct. Because cognitive function is intact it is sometimes referred to as a pseudo coma state; MeSH). Were there still refractory symptoms, how were they treated by the health care team? Was the palliative sedation therapy changed from light into deep? And why was this change made? Were there adverse events due to the palliative sedation? | |
|  | o   Consciousness |  |  |
|  | o   Lock in syndrome |  |  |
|  | o   Refractory symptoms |  |  |
|  | o   Restlessness |  |  |
|  | o   Relief distress for the wife/family | The palliative sedation does not have only an effect on the patient but can also affect the relatives, e.g. relieve of distress for the relatives. For the present subcode the focus is on the short term-effect of PS on the relatives | |
|  | Adverse events/ unexpected events/ physical changes patient related with dying | Adverse events could be dyspnoea, decreased respiratory rates, decreased oxygen saturation, suffocation and paradoxical agitation were all cited in the systematic review of Maria Arantzamendi et al.. Adverse events or often not reported in studies. Another event that could be seen as an adverse event is that the patient dies very shortly after applying PS (e.g., within an hour). While a person dies physical changes occur for instance getting blue spots on the body. It is plausible that a relative will not know which changes is related with dying and which is an adverse outcome. Both will be included in the present code. | |
|  | Suboptimal sedation | No clear definition with regard to suboptimal sedation exists. In a study of Pype et al., suboptimal sedation was defined using two criteria: the time span until deep sleep being more than one and a half hour, and/or three or more awakenings after deep sleep was reached. At the moment a nurse/physician/relative indicates that the application of the sedation was suboptimal/problematic it should be coded as suboptimal sedation. | |
| alternatives therapies |  | Alternative therapies could be an option. On the one side the HCP could have the feeling that alternative options would have been more appropriate instead of PS for the patient. It could also be that alternative options during the care were tried, but did not relieved the patient symptoms. This indicates that the alternative options were taken into consideration to determine whether PS was the option of last resort. | |
| Couldn’t communicate with the patient | Loss communication patient |  | |
| Sedation in conflict with religious/ cultural or moral beliefs | Conflict religious/moral |  | |
| Feeling the burden of responsibility of the decision |  | This can be the burden of the responsibility or the burden of not have taken the responsibility of the decision. | |
|  | Emotional burden |  | |
|  | o   Guilt |  | |
|  | o   Powerlessness |  | |
|  | physical |  | |
|  | o   exhaustion |  | |
|  | Uncertainty end-of-life wishes patient | No discussions were held with regard to advance care planning and end-of-life decisions before the initiation of PS | |
| Preparedness about the condition – | preparedness | The family member/relative was not prepared for the change in the physical condition of the patient, was not ready to say goodbye. The focus is on the mental aspect of being prepared about the change. One recognizes and accept the situation. | |
| opportunity to say goodbye |  |  | |
| Dying with dignity | Dying with dignity |  | |
| a good death | a good death |  | |
| A worthy goodbye | A worthy goodbye |  | |
| The idea of a solution for the suffering of a patient |  | This is more at the introduction of PS, people are glad to hear there is a possible solution to end the suffering. This does not suggest the PS is effective. Although this is more for the family members and not for the HCP | |
| Artificial food and liquid | Nutrition and hydration |  | |
| (psychological) Distress | Patient  relative | -The National Comprehensive Cancer Network deﬁned distress as a “multifactorial, unpleasant emotional experience of a psychological (cognitive, emotional), social, and/or spiritual nature that may interfere with the ability to cope effectively with cancer, its physical symptoms, and its treatment.” Thus, distress can be considered to range from normal fears, worry, and sadness to disabling problems such as clinical depression, generalized anxiety, panic, isolation, or a spiritual or existential crisis (The NCCN Guideline for Distress Management: A Case for Making Distress the Sixth Vital Sig, Distress in Older Patients With Cancer). | |
|  | Timing of PS | -The timing of PS can also be distressing. If the relatives/HCP indicate that PS should have been earlier and that therefore the patient suffered unnecessary. | |
|  | HCP | Negative emotional state characterized by physical and/or emotional discomfort, pain, or anguish | |
| Team related – discord within the team | Discord within team | A lack of coherence between the health care professionals in the choices made. For instance, one does not agree whether the symptoms are refractory or not (there were differences in opinion with regard intermittent or continuous sedation, the intention of the sedation). According to previous research in some cases palliative sedation is applied with the intention to hasten death. This could distress the other health care professionals. This discord could be noticed by the relatives and could affect how they experience PS. | |
| interactions with the HCP | Negative interaction: Relatives experience difficulties with HCPs | Relatives can experience the communication with HCPs as difficult, can feel some pressure to agree with the PS of a relative. | |
|  | Positive interaction | HCP were accessible, time to talk and give information, … | |
| Interaction patient and HCP | Positive interaction: a good connection, bounding, understanding the patients will, good communication |  | |
|  | Negative interaction |  |  |
| Interactions between patient and family | Positive interaction |  | |
|  | Negative interaction: Tensions within the patient’s family/ relatives | Tension within the family, family members/relatives not talking with each other, the difficult relation between the patient and his family members/relatives | |
|  | A young child |  | |
|  | disconnection |  | |
|  | compagneship | The interaction between patient and relatives does not indicate that the patient must be conscious. This means that even during the sedation there can be interaction between patient and relatives, e.g. holding hands, playing music, | |
| Circumstances | Environment not conducive – privacy, busy ward etc | For instance, the extra measures of the corona pandemic which extra distress the health care professionals, personal conflicts/tensions between health care professionals. The situation in which the palliative sedation was applied was not conducive. For instance, there was a lot of noise in the hall, many people were walking around in the hall, or there was not a private room (lack of privacy). | |
|  | Time of year | The time of the year can be distressing, for instance religious holidays such as Christmas/Pesach/Ramadan: people are spending time with family, or perhaps summer vacation: many people are on holiday and therefore much fewer employees are available. | |
| Satisfaction of the treatment of the sedated patient | Satisfaction of the treatment of the sedated patient |  | |
|  | o   Technical skills | Technical quality: Competence of providers and adherence to high standards of diagnosis and treatment (thoroughness, accuracy, unnecessary risks, making mistakes, ...) | |
|  | o   Interpersonal manner | Interpersonal manner: Features of the way in which providers interact personally with patients (concern, friendliness, courtesy, respect, rudeness, sensitivity, tact, honesty, confidence, presence, welcoming, ...) | |
|  | o   Communication skills | Communication skills: Aptitudes in eliciting information not only on physical but also on psychosocial aspects, in providing information on subjects of patients’ concerns | |
|  | o   Coordination | Coordination: Management of the care provided by a multi-disciplinary team and the operational exchange of oral or written information between them | |
|  | o   Continuity | Continuity: Sameness of providers | |
|  | o   Environment | Physical environment: Physical features of the care setting and other “hotel-like” aspects of care or amenities | |
|  | o   Waiting time | Waiting time: Organizational feature of care, such as waiting for medical test results, waiting time before a consultation | |
| (No) comparability with other cases | Not knowing what to expect (in case of family/relative) | for a family member/relative the experience of PS is often new. One can often not compare with other experiences. If an adverse event occurs, this will be experienced as unexpected, while for a HCP this will not be the case. | |
|  | o (Lack of) information |  |  |
| Factors external to PS |  | Organisational factors for instance will the grandchildren or great grandchildren get the opportunity to visit a last time the patient, or a child or grand-child is on vacation or away abroad and is not able to say goodbye in the first 24 hours, organisational factors with regard to the future funeral, financial issues that need to be made in advance before the patient dies. Preparations needed/concerns for after the patient dies, for instance what with the remaining partner is he/she able to live alone at home | |
| Coping with the PS procedure |  | Coping is defined as the thoughts and behaviours mobilized to manage internal and external stressful situations.  Coping is generally categorized into four major categories which are: | |
|  | o Problem-focused | Problem-focused, which addresses the problem causing the distress: Examples of this style include active coping, planning, restraint coping, and suppression of competing activities. | |
|  | o Emotion-focused | Emotion-focused, which aims to reduce the negative emotions associated with the problem: Examples of this style include positive reframing, acceptance, turning to religion, and humour. | |
|  | o Meaning-focused | Meaning-focused, in which an individual uses cognitive strategies to derive and manage the meaning of the situation | |
|  | o Social coping | Social coping (support-seeking) in which an individual reduces stress by seeking emotional or instrumental support from their community. | |
| Care for the family | o Care before death patient |  | |
|  | o Bereavement care |  | |
| Communication style |  | the characteristic way a person sends verbal, and nonverbal signals in social interactions denoting (a) who he or she is or wants to (appear to) be, (b) how he or she tends to relate to people with whom he or she interacts, and (c) in what way his or her messages should usually be interpreted.” (De Vries et al., 2009, p. 179).  Different communication style models exist. I explain the communication style inventory (CSI). To summarize, the CSI is meant to represent six behavioural communication style dimensions:  1) Expressiveness includes Talkativeness, Conversational dominance, Humour and Informality,  2) Preciseness includes includes Structuredness, Thoughtfulness, Substantiveness and Conciseness,  3) Verbal Aggressiveness includes Angriness, Authoritarianism, Derogatoriness and Nonsupportiveness,  4) Questioningness includes Unconventionality, Philosophicalness, Inquisitiveness, Argumentativeness,  5) Emotionality includes Sentimentality, Worrisomeness, Tension, Defensiveness,  6) Impression Manipulativeness includes Ingratiation, Charm, Inscrutableness and Concealingness | |
| others |  |  | |
